# Complicated traumatic pulmonary pseudocyst: a case report

**DOI:** 10.1016/j.tcr.2025.101221

**Published:** 2025-07-10

**Authors:** Ali Hossein Samadi Takaldani, Rona Jannati, Amirpasha Mansour

**Affiliations:** a Department of Internal Medicine (Pulmonary Division), School of Medicine, Ardabil University of Medical Sciences, Ardabil, Iran; b Department of Internal Medicine, School of Medicine, Ardabil University of Medical Sciences, Ardabil, Iran; c Department of Anesthesiology and Pain Medicine, School of Medicine, Tehran University of Medical Sciences, Tehran, Iran

**Keywords:** Pulmonary pseudocyst, Trauma, Hemothorax, Lesion

## Abstract

A traumatic pulmonary pseudocyst (TPP) is an uncommon lung lesion that can occur due to blunt chest trauma. This condition is usually harmless and doesn't require medical treatment, but in rare cases, it is complicated by rupture of the lesion and hemothorax. We present the case of a 43-year-old man who fell from a height and suffered chest trauma. Imaging revealed a mass-like lesion and hemothorax, but after ruling out other diagnoses, he was diagnosed with complicated traumatic pulmonary pseudocyst. Over six months, the mass-like lesion shrank and disappeared entirely without any remaining scars. Proper diagnosis and differentiation from other lung lesions are essential in cases of TPP. With conservative management and regular follow-up, the prognosis is generally reasonable.

## Introduction

Traumatic pulmonary pseudocyst (TPP) is an uncommon pathology seen in patients who suffer from blunt chest trauma and pulmonary parenchymal injuries. It has been reported to occur in 3 to 8.3 % of all chest traumas [1,2]. TPP manifests as a cavitary lesion that lacks an epithelial lining, thus setting it apart from other etiologies of parenchymal cavitary lesions, such as tuberculosis. This feature enables a differential diagnosis and facilitates accurate identification of the condition. These lesions often resolve spontaneously and do not require specific treatments. However, surgery may be necessary for complicated lesions [3,4].

Here, we present a pulmonary parenchymal injury following a fall that produces a mass-like lesion. After ruling out other diagnoses, the lesion leads to a TPP diagnosis. The lesion resolves spontaneously, leaving no parenchymal scar behind.

## Case presentation

A 43-year-old male was referred to the hospital for shortness of breath and mild left hemithorax pain. He noticed his breathing difficulties a week ago, which initially made him walk slower than others. However, his condition worsened, and he was unable to walk >100 m on level ground (mMRC II-III) at the time of referral. He also mentioned Left hemithorax pain following deep breaths without coughing. He also noted that he had fallen from a height of 3 m about a month ago. During the incident, he fell on his chest, causing fractures on three points of the ribs. The ribs affected were the seventh to ninth ribs of the left hemithorax on the midaxillary line. At the time of the incident, imaging during admission showed no complications such as hemothorax or pneumothorax.

In examination, his vital signs were normal, and he was afebrile. In observation, a few healing abrasions were evident. Also, he had a symmetrical chest expansion. The patient had local tenderness on palpation in the left chest wall, especially on the midaxillary line of the seventh to ninth ribs. On lung auscultation, decreased lung sounds were auscultated in the left basal region of the chest. In the chest CT scan without enhancement, moderate left hemithorax pleural effusion with passive sub-pleural collapse, a 35*55 mm consolidation in the Inferior lingula segment of the left lung, and evidence of rib fractures in the left chest wall were reported. Measuring the radiofrequency of the lesion in a CT scan showed the mean of 12 Hounsfield units, favoring the fluid nature of the fluid aggregation in the cavitary lesion [5]. Considering the prior history of trauma, traumatic pulmonary pseudocyst was also in the differential diagnosis. As the patient had a one-sided pleural effusion, to rule out other diagnoses such as lung malignancies, infections, and hemothorax, during the diagnostic procedure, a thoracocentesis was carried out, and a red fluid with an exudative nature was observed. The hematocrit level of the fluid was found to be higher than 50 % of the hematocrit level of the blood sample taken at the same time. This finding indicated that the patient was suffering from hemothorax. Afterwards, a surgery consult was requested, and a chest catheter was inserted with the guidance of ultrasonography. Cytology and culture of fluid were negative (Table 1).

**Table 1:** Laboratory results of patient's blood test.

	Reference value	Admission	Discharge
WBC (cu/mm)	4000–10,000	5800	6200
Hb (g/dl)	12–16	12.8	12.7
MCV (fl)	80–100	86	85
HCT (%)	39–52	39.1	37.8
Platelets (10^6/ml)	150–450	193	150
PTT (s)	30–35	30	43
INR (Index)	1–1.4	1.2	1
Urea (mg/dl)	15–45	26	–
Creatinine (mg/dl)	0.5–1.4	0.96	–
AST (IU/L)	5–40	14	–
ALT (IU/L)	5–40	8	–
ALP (IU/L)	64–306	221	–
ESR (mm/h)	<20	8	–
CRP	Negative	Negative	–
Pleural sugar (mg/dl)	>60	86	–
Pleural LDH (IU/L)		470	–
Pleural protein (mg/dl)	<35	36	–
Pleural HCT (%)	<50 % of blood HCT	36.3	–
LDH (IU/L)	0–500	308	–
Total protein (mg/dl)	60–80	54	–

WBC: white blood cells; HB: hemoglobin; HCT: hematocrit; MCV: mean corpuscular volume; MCH: mean corpuscular hemoglobin; HCT: hematocrit; LDH: lactate dehydrogenase; CRP: C-reactive protein; ESR: erythrocyte sedimentation rate; INR: international normalized ratio; PTT: partial thromboplastin time; AST: aspartate aminotransferase; ALT: alanine aminotransferase; ALP: alkaline phosphatase.

**Fig. 1:**
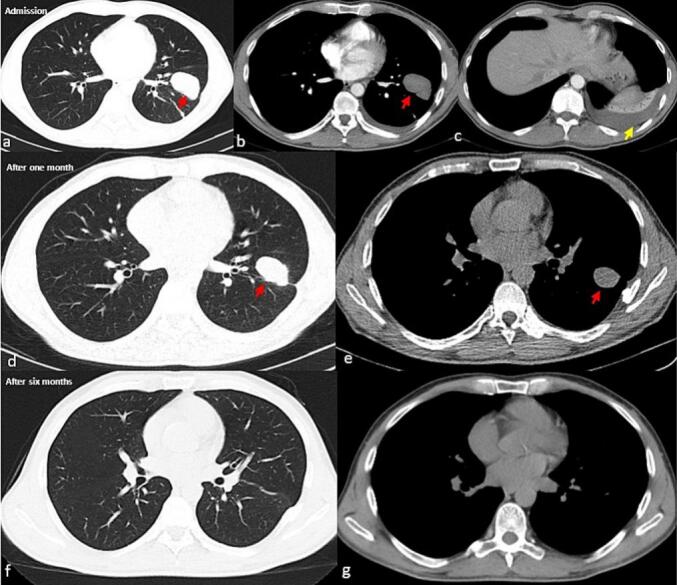
The patient chest CT scans without contrast at admission, one month after admission, and six months after admission. Figures a, d, and f show the parenchymal view, while figures b, c, e, and g present the mediastinal view of the patient's chest CTs. The imaging reveals the spontaneous resolution of traumatic pulmonary pseudocyst (red arrow) and the complication of pseudocyst with hemothorax (yellow arrow).

A cardiology consultation was requested, and electrocardiographic and echocardiographic studies were normal. AFB smear and sputum culture were requested to rule out tuberculosis, and the results returned negative. He was treated with meropenem ampule 1 g thrice daily, vancomycin ampule 1 g twice daily, and conservative treatments. After two days, the fluid was discharged entirely, and the catheter was removed. Two days later, he was discharged with SpO2 of 96 % and without shortness of breath and was advised to come to the pulmonology clinic one month later. One month later, a follow-up CT scan showed the lesion had shrunk, and after six months, the lesion had resolved entirely (Fig. 1). The spontaneous resolution of the lesion confirmed the TPP diagnosis.

## Discussion

The most common injuries to the lungs resulting from blunt trauma are contusions and lacerations [6]. Pulmonary lacerations are severe injuries caused by tears in the lung parenchyma. These tears can lead to pneumothorax or hemothorax [7].

Traumatic pulmonary pseudocysts (TPPs), also known as traumatic pneumatoceles, are cavitary structures formed due to high-energy traumas like motor vehicle accidents and falls [8]. TPPs are typically observed in children and young adults aged <30 years old [9]. Several mechanisms could lead to the formation of TPP. However, most researchers agree that pseudocysts develop due to shearing forces that cause pulmonary laceration accompanied by the accumulation of air or fluid in the tissue. TPPs could be single or multiple, and although they can appear in any part of the lung, the most common sites are the lower lobes [4]. Lesions can appear within 24–48 h after trauma and change in size and shape over time, distinguishing TPP from other cavity lesions [10]. The typical symptoms of TPP include dyspnea, hemoptysis, chest pain, cough, fever, or sometimes no symptoms. However, these symptoms are not specific to TPP and are typically associated with pulmonary parenchymal injury [3]. Our patient presented with shortness of breath due to complicated TPP with hemothorax. Fortunately, after the insertion of a lung catheter, the condition alleviated. The chest pain was the result of multiple rib fractures which occurred during the incident. Although this condition typically affects people under 30, our patient was 43 years old and had a single TPP filled with fluid [9].

It's essential to distinguish TPP from other cavitary lung lesions. Differential diagnoses of TPP include lung abscess, bronchial cyst, tuberculosis, mycosis, and neoplasm [11]. The diagnosis of TPP is typically determined by evaluating the patient's trauma history and radiologic findings. The diagnosis of lung lesions has been addressed through various modalities. In recent years, artificial intelligence has emerged as a promising approach in this field [12,13]. However, in cases of TPP, a chest radiography may show the lesion, but a CT scan is the preferred imaging modality. In CT, the presence of thin-walled single or multiple cysts and air space consolidation in the surrounding parenchyma has been suggested as a diagnostic indicator [14]. To confirm the diagnosis, follow-up radiological imaging can be performed to show the resolution of the lesions over time [4]. The typical course of TPP is usually benign and resolves on its own over a few months [15]. In our case, the lesion took six months to completely disappear from the imaging.

TPPs are usually not treated until they become complicated [16]. The conservative treatment for TPP involves pulmonary hygiene, ruling out infectious causes, and regular radiological imaging to check for spontaneous resolution. However, surgery may be necessary if the TPP is complicated with infection, bleeding, or rupture into the pleural space or does not resolve on its own [4]. In our case, the pleural effusion appears to have occurred due to a rupture of the TPP. The high hematocrit levels favored a hemothorax diagnosis, so the pleural catheter was implanted.

Our case was a middle-aged man who presented to the hospital with complicated TPP with hemothorax, which led to pleural catheter insertion. Afterward, the remaining TPP was resolved spontaneously after six months without leaving a scar.

## Conclusion

Traumatic pulmonary pseudocyst is an uncommon benign lung lesion that occurs as a result of blunt chest trauma. It is characterized by the formation of cavitary structures without an epithelial lining. TPPs typically resolve spontaneously and do not require specific treatment. However, in complicated cases, surgical intervention may be necessary. Despite the scarcity of the appearance of TPP in general, the appearance of this lesion in a middle-aged patient and the complication of the case with rupture of the cyst and hemothorax formation is what makes this case uncommon. It is important to note that in patients with chest trauma, a mass-like lesion with fluid features may indicate TPP as a possible diagnosis after other conditions have been ruled out.

## Ethical approval and informed consent statements

Written informed consent was obtained from the patient to publish this case report and any accompanying images. A copy of the written consent is available for review by the Editor-in-Chief of this journal.

## CRediT authorship contribution statement

**Ali Hossein Samadi Takaldani:** Writing – original draft. **Rona Jannati:** Writing – review & editing. **Amirpasha Mansour:** Writing – review & editing.

## Consent to participate

Written informed consent was obtained from the patient for his participation in this case report. A copy of the written consent is available for review by the Editor-in-Chief of this journal.

## Ethical approval

Our institution does not require ethical approval to report individual cases.

## Funding

This article was prepared without any support or funding and.

## Declaration of competing interest

The authors declare that they have no competing interest to disclose.

## Data Availability

The study data is available from the corresponding author upon a reasonable request.
